# Histopathological, Immunohistochemical, Molecular and Genetic Biomarkers in Differentiated Thyroid Cancer

**DOI:** 10.3390/cancers17172869

**Published:** 2025-08-31

**Authors:** Mousa A. Al-Abbadi, Dunia Aburizeg, Husam Abuawad, Hala Alzaghloul, Omar Sqour, Bilal Azab, Tala Qudisat, Ali M. Alabbadi, Ayman Mismar, Malik Eid Juweid

**Affiliations:** 1 Department of Histopathology, Microbiology and Forensic Medicine, School of Medicine, University of Jordan, Amman 11942, Jordan; d_aburezeq@ju.edu.jo (D.A.); hsa8221664@ju.edu.jo (H.A.); bazab@phoenixchildrens.com (B.A.); 2 School of Medicine, University of Jordan, Amman 11942, Jordantal0208672@ju.edu.jo (T.Q.); 3 Division of Pathology and Laboratory Medicine, Phoenix Children’s Hospital, Phoenix, AZ 85016, USA; 4 Department of Child Health, College of Medicine, University of Arizona, Phoenix, AZ 85004, USA; 5 Department of Radiology and Nuclear Medicine, School of Medicine, University of Jordan, Amman 11942, Jordan; 6 Department of General Surgery, Jordan University Hospital, Amman 11942, Jordan

**Keywords:** thyroid cancer biomarkers, review, histopathological immunohistochemical molecular, and genetic

## Abstract

Differentiated thyroid cancer (DTC) is the most prevalent endocrine malignancy in the world. Accurate diagnosis and prognostication are essential for optimizing its treatment and improving patient outcomes. This narrative review explores the diagnostic and prognostic histopathological, immunohistochemical, molecular, and genetic biomarkers in DTC, emphasizing their role in risk stratification and personalized management. Histopathological biomarkers, including tumor size, extrathyroidal extension, lymphovascular invasion, and aggressive subtypes (e.g., tall cell, hobnail, and insular variants), correlate with poor prognosis. Additionally, genetic alterations such as *BRAF*:p.V600E, *RAS* mutations, *TERT* promoter mutations, and *RET*/*PTC* rearrangements provide molecular insights into tumor progression and therapeutic response. Some of these molecular/genetic mutations have surrogate proteins that are feasible for immunohistochemical analysis, providing faster and cost-effective alternatives. Advances in next-generation sequencing have further refined risk stratification, facilitating precision medicine approaches. Future research should focus on validating novel biomarkers and developing targeted therapies to improve patient outcomes.

## Simple Summary

Thyroid cancer is the most common endocrine malignancy, and its incidence is on the rise worldwide. The burden of the disease on each nation’s health costs is significant. The majority of these neoplasms are considered low-grade carcinomas, where papillary thyroid carcinoma (PTC) is the most frequent, followed by follicular and oncocytic thyroid carcinoma (FTC and OTC, respectively). These three are known as differentiated thyroid carcinoma (DTC). Fine-needle aspiration (FNA) of thyroid lesions is essential in the preoperative evaluation of these nodules. The main aim of thyroid FNA is to establish an accurate diagnosis to determine which patients require surgery and to prevent unnecessary procedures. Recent advances in FNA techniques, standardization of reporting systems, and the availability of immunohistochemical and molecular testing on FNA material allow treating clinicians to determine the best triaging approach for these patients. The application of these tests may have diagnostic or prognostic value and sometimes both. In this narrative review, we provide a thorough overview of these biomarkers in DTC. The current standard of care in the utilization of these biomarkers and the difficulties we face in their implementation are discussed. Future expectations in the field of DTC biomarkers are also presented.

## 1. Introduction

Thyroid carcinomas are the most common endocrine malignancies worldwide. Among these, papillary thyroid carcinoma (PTC), follicular carcinoma (FTC), and oncocytic carcinoma (OTC) are together considered “differentiated thyroid carcinoma (DTC)” and make up over 90% of thyroid cancers [1,2]. The other much less common malignancies include medullary thyroid carcinoma (MTC), anaplastic thyroid carcinoma (ATC), and primary thyroid lymphoma [1,2].

It is worth mentioning that DTC in pediatric patients, in many aspects, is similar to their adult counterparts [3]. However, a few distinct and unique differences impact their clinical behavior and response to treatment. Guleria et al.’s review concluded that in pediatric DTC, chromosomal alterations are more common than point mutations compared to DTC in adults, where point mutations are more common. However, they alluded that larger studies are needed since DTC in children is less common than in adults [3].

Overall, the incidence of DTC has been on the rise; however, most patients are performing increasingly well with decreasing mortality rates and recurrence [4,5]. This is attributed to earlier detection and improved treatment [4,5]. This was also attributed to the introduction of standardized systems for evaluating and reporting thyroid lesions by fine-needle aspiration (FNA), such as The Bethesda System for Reporting Thyroid Cytopathology (TBSRTC) and the British Thy system and others [6,7]. The implementation of “risk of malignancy” and subsequent triage recommendations using objective criteria and evidence-based practice approaches strengthened the worldwide acceptance of these reporting systems [8]. The most recent World Health Organization (WHO) classification of thyroid neoplasms utilized these diagnostic approaches in addition to guidelines for treatment and prognosis with special emphasis on molecular studies [8].

It is worth mentioning that OTC, which used to be called Hürthle cell carcinoma, is now considered a separate entity and was removed from the FTC group [8]. This recent change is due to the recognition of unique molecular abnormalities and different clinical behavior in OTC [8].

**Figure 1:**
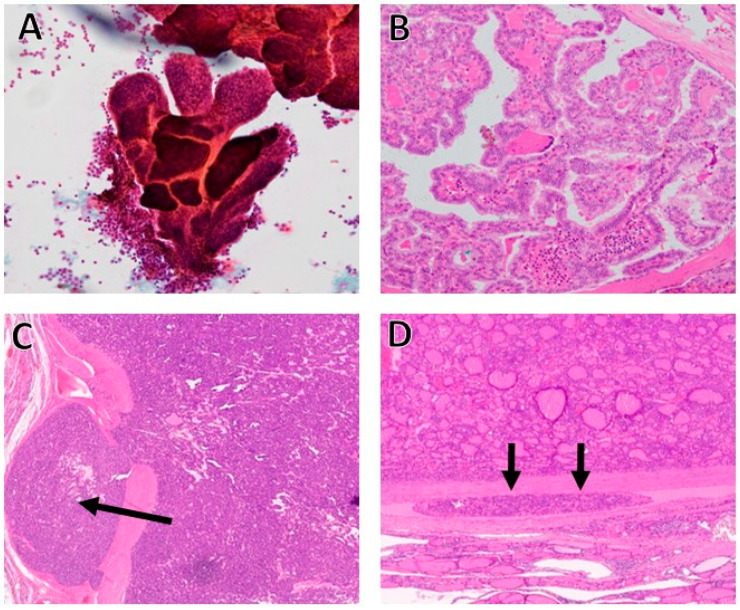
(**A**): Low-power view of papillary thyroid carcinoma (PTC), a diagnosis that can be made easily on fine-needle aspiration material. Notice the high cellularity, papillae, and scarcity of colloid. At higher power, the characteristic PTC nuclear features were clearly identified. The diagnosis on the FNA material utilizing the standards of the Bethesda system was category 6, positive for malignancy, PTC (Papanicolaou stain, 200×). (**B**) Medium power view of histologic section of the same patient in image A exhibiting classic variant of PTC with papillary formation and the characteristic nuclear features of PTC (Hematoxylin and Eosin stain, 200×). (**C**) Medium power view of follicular carcinoma where the follicular-patterned neoplasm invades the pink capsule and “mushrooming” through it (black arrow), and in (**D**) the tumor is seen invading the blood vessel (black arrows). Capsular invasion and vascular invasion are required criteria to justify the diagnosis of carcinoma; two features that can only be identified on histological sections and are impossible to identify on aspiration cytological smears.

**Figure 2:**
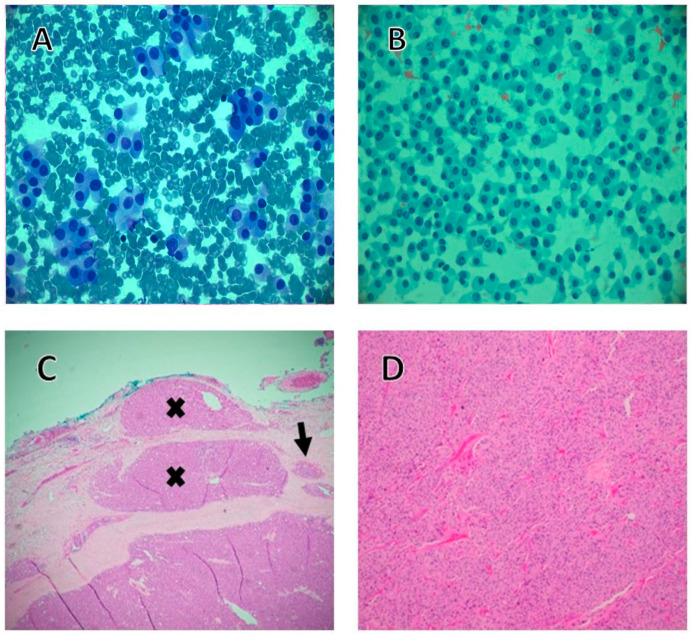
(**A**) Diff-Quik-stained aspirate smear of a thyroid nodule revealing predominance of thyroid epithelial cells exhibiting granular, abundant cytoplasm in multiple clusters and scarcity of colloid (Diff-Quik stain, 400×). (**B**) Smears from the same patient but with Papanicolaou stain showing similar features (Papanicolaou stain, 400×). The cytological diagnosis based on the standards of the Bethesda system was correctly interpreted as “Follicular neoplasm/suspicious for follicular neoplasm” with oncocytic features, which corresponds to the Bethesda system category 4. Akin to follicular neoplasms, differentiating oncocytic adenoma from oncocytic carcinoma requires thorough histological evaluation documenting invasion to capsule and/or vessels as demonstrated in C. (**C**) On thyroidectomy, the tumor was completely oncocytic, composed of oncocytes (used to be known as Hürthle cells) with extensive capsular invasion (black X) and in addition to vascular invasion (black arrow) as shown in this microscopic photograph confirming the diagnosis of carcinoma (Hematoxylin and Eosin stain, 40×). (**D**) Medium power view highlighting the oncocytic features of this tumor in the form of large, abundant eosinophilic and granular cytoplasm on routine Hematoxylin and Eosin stain, hence the description “oncocytic”, which is known to be due to numerous mitochondria. In addition, significant nuclear atypical features are noted, which are characteristic of oncocytic neoplasms, such as hyperchromasia and nuclear pleomorphism, which are better appreciated on higher power (Hematoxylin and Eosin stain, 40×).

The routine evaluation of thyroid nodules includes clinical examination, imaging studies, and FNA, which are routinely performed under ultrasound image guidance. The interpretive accuracy of FNA of thyroid nodules is very high for PTC (Figure 1A,B) although it is less so for follicular and oncocytic neoplasms [8]. It is therefore well-known and well-accepted that the definite diagnosis of FTC and OTC requires thorough histopathological examination (Figure 1C,D and Figure 2) of the resected specimens [9].

This narrative review focuses on the diagnostic and prognostic significance of histopathological parameters, immunohistochemical (IHC) stains, and molecular and genetic biomarkers in DTC, emphasizing their role in diagnosis, risk stratification, and personalized management.

Noteworthy, this review utilized, to the best of our knowledge, the most relevant literature evidence. Nevertheless, evaluating the quality and strength of each reference was beyond the scope of this review (as discussed in Section 6).

## 2. Epidemiological and Clinical Parameters

Some studies attempted to correlate specific dietary habits, including tea, coffee, and fish, with thyroid cancers; however, statistical analysis failed to back such claims [10,11].

Some benign thyroid disorders, such as Hashimoto’s thyroiditis, Graves’ disease, and multinodular goiter, are associated with a higher risk for developing thyroid carcinoma, with PTC being the most common malignancy in these patients [3,12,13].

Factors that are associated with poor prognosis common to both PTC and FTC include obesity, family history of thyroid cancer, and advanced age at diagnosis [1,14,15].

The presence of bone metastasis in DTC carries a higher mortality, especially in older patients who have concurrent lymph node metastases [16]. Of the DTC subtypes, OTC tend to have higher rates of metastases at presentation [16,17].

Even with the presence of poor prognostic factors, DTC is still considered to have favorable outcomes, especially in the pediatric age group [18,19]. Table 1 shows the various clinical parameters determining prognosis in DTC.

## 3. Histopathological Parameters

### 3.1. Papillary Thyroid Carcinoma

PTC is the most prevalent endocrine malignant neoplasm, and consequently underwent extensive literature evaluation for its cytomorphological, histological, IHC, and molecular and genetic characteristics, with many studies addressing the relationship of these features with prognosis [2].

Traditionally, some histologic variants of PTC were associated with worse prognosis and more aggressive disease, namely, the tall cell, hobnail, columnar, diffuse sclerosing, solid, trabecular, and insular variants [8]. Moreover, Bournaud et al. linked some specific histological features with extra-thyroidal extension and telomerase reverse transcriptase (*TERT*) mutations; both of which are well-known poor prognostic parameters [21].

PTC shows a worse prognosis when accompanied by extra-thyroidal extension compared to its encapsulated counterpart, as evidenced by higher rates of central lymph node metastasis (stage: N1a) shown by Jin et al. [20]. However, for the involvement of lateral lymph nodes (stage: N1b), only gross extra-thyroidal extension was associated with N1b status. There was no significant difference between encapsulated variants and those with minimal extra-thyroidal extension with respect to this status [20]. This is also true for the solid variant of PTC (solid/trabecular/insular growth), which is not associated with a change in prognosis by itself, but its encapsulation improves its prognosis, while the presence of invasion to the surrounding tissue through its capsule is considered a poor prognostic sign [22].

Other histopathological parameters associated with worse prognosis include tumor size, the presence of distant metastases, lympho-vascular invasion, and lymph node metastases [23]. However, multifocal disease remains a controversial topic as it is considered a poor prognostic factor in the latest WHO classification, but recent studies are divided on this issue [8]. For example, in 2017, Wang et al. reported that multifocal carcinoma has no clinical prognostic significance in patients with an established diagnosis of PTC [23,24]. For lymph node metastases, Vrachimis et al. noted that a stage N1a does not necessarily result in a worse prognosis, while stage N1b definitely does, which is in line with the WHO consideration of N1b but not N1a status as a poor prognostic indicator [8,14,15,23,24,25,26,27]. Table 2 summarizes the most important histopathological parameters.

### 3.2. Follicular Thyroid Carcinoma

Second only to PTC, FTC prevalence and disease burden are high compared to other endocrine tumors, and becomes even more prevalent in the elderly [2,28].

The recent WHO classification states that for a definite diagnosis of FTC, capsular and/or vascular invasion are required (Figure 1C,D) [8]. This means that such a diagnosis can only be established on excised thyroid lobes. Therefore, establishing an FTC diagnosis on FNA cytological smears is impossible. As such, researchers have been searching for less invasive markers to differentiate follicular adenoma (FA) from carcinoma [8].

A follicular-patterned thyroid neoplasm lacking vascular invasion but with definite histological evidence of focal capsular invasion is termed “minimally invasive follicular thyroid carcinoma” [8]. This entity is shown to have a better prognosis and can be treated more conservatively [29]. On the other hand, follicular carcinomas with vascular invasion have a much higher risk of complications and exhibit worse prognosis and consequently require more aggressive treatment plans. Matsuura et al. proposed a cutoff of two or more foci of vascular invasion as a risk factor for mortality and recurrence compared to those with a single focus of vascular invasion [30].

Sawant et al., who compared cohorts of DTC from the United Kingdom to other parts of the world, reported a seemingly worse prognosis in certain geographical areas, as they seem to be associated with higher rates of lymph node involvement and/or distant metastases [31]. Table 3 summarizes the most important histological parameters of FTC and their prognostic significance.

### 3.3. Oncocytic Thyroid Carcinoma

Previously considered a subtype of FTC, OTC is now considered a separate entity according to the latest fifth edition of the WHO classification of tumors [8]. To establish such a diagnosis, the lesion must comprise at least 75% oncocytic cells with evident capsular and/or vascular invasion akin to their follicular counterpart. OTC is generally considered more aggressive than the other DTC subtypes [8,32,33]. As with FTC, the presence of vascular invasion and the number of such foci are the main prognostic factors in OTC. Tumors with only capsular invasion and no vascular invasion are termed “minimally invasive OTC” and have an excellent prognosis, while tumors with one–three foci of vascular invasion are considered “invasive OTC” (Figure 2). Those with four or more foci are termed “widely invasive,” carrying a very poor prognosis [17].

Very few studies addressed the histopathological parameters of OTC. However, more studies are focused on the molecular and genetic features (*vide infra* in the section on molecular and genetic features).

## 4. Immunohistochemical (IHC) Biomarkers in Differentiated Thyroid Carcinoma

### 4.1. IHC in Papillary Thyroid Carcinoma

IHC can reliably detect thyrocytes among other cell types present in a thyroid sample, such as connective tissue cells, C-cells, and cells from metastatic tumors to the thyroid. IHC utilizes antibodies directed against molecules relatively specific to thyrocytes, such as thyroglobulin (Tg), thyroid transcription factor 1 (TTF1), which modulates the expression of the thyroid peroxidase (TPO), and paired box gene 8 (*PAX8*). However, because these markers are expressed in all follicular-derived benign and malignant thyroid cells, it is difficult to distinguish between benign and malignant lesions using these markers [34,35]. Tg, a protein synthesized by thyrocytes, has been shown to be profusely expressed in follicular-derived thyroid cancers, with positive IHC staining of Tg demonstrated in DTC. Therefore, IHC staining for Tg is considered an organ-specific marker and consequently is particularly useful to confirm the thyroid origin of any atypical epithelial cells in cytopathological and histological samples [36,37]. One other marker that is widely used in routine IHC as an organ-origin marker is PAX8. It is an antibody against one of the members of the paired box gene family transcription factors, which can be easily interpreted for its positivity as it is a nuclear stain [38]. Although it confirms thyroid follicular cell origin when it is positive, it lacks specificity since it can be expressed in a few other organs, such as renal and Müllerian epithelial cells. Therefore, it is usually utilized in conjunction with other markers to enhance specificity [38].

**Figure 3:**
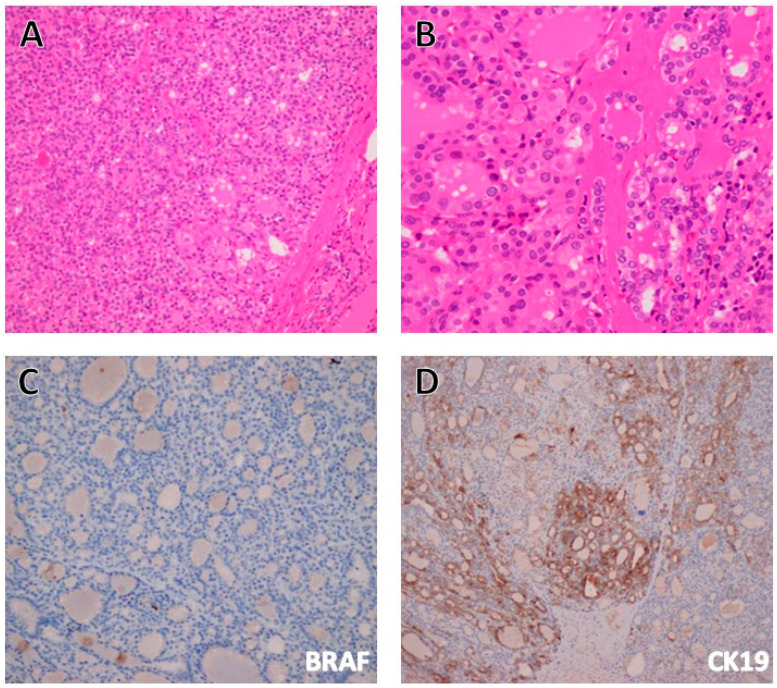
(**A**) A medium power view of the total thyroidectomy specimen showing a small nodule that is encapsulated (notice pink capsule at the bottom right corner). This nodule was aspirated before, with a cytological interpretation of “Atypia of undetermined significance” twice, corresponding to the standardized Bethesda system category 3. The morphological appearance is of a follicular pattern lesion, with mild and soft PTC nuclear features (Hematoxylin and Eosin, 200×). (**B**) Higher power view demonstrating the follicular pattern (small back-to-back follicles) and the soft PTC nuclear features in the form of nuclear clearing, occasional nuclear grooves, mild overlapping, and minimal pleomorphism (Hematoxylin and Eosin, 400×). Given the aforementioned histopathological parameters, the differential diagnosis is between encapsulated follicular variant of PTC or noninvasive follicular thyroid neoplasm with papillary-like nuclear features (NIFTP). This differential diagnosis is one of the challenging issues in surgical pathology, despite the fact that both behave in an indolent and almost benign fashion. We utilized IHC for BRAF, CK19 and Galectin-3 in this case, trying to clear up the differential diagnosis. The IHC for BRAF was completely negative (as shown in (**C**)) but focal staining for CK19 was identified (**D**). Galectin-3 immunostaining was completely negative. Therefore, the diagnosis of NIFTP is favored.

Nevertheless, some markers are known to be promising since they are predominantly expressed in malignant cells. These include Hector Battifora mesothelial epitope-1 (HBME-1), cytokeratin 19 (CK19), and Galectin-3, which are the most widely recognized markers that are expressed in over 95% of PTC, with very low expression in benign thyroid neoplasms [39]. Therefore, utilizing these markers becomes very helpful to differentiate PTC from benign lesions [39]. This diagnostic challenge is well-known when dealing with follicular-patterned thyroid neoplasms when the nuclear features of PTC are very soft or weak in nature (Figure 3).

While both CK19 and Galectin-3 have similar sensitivity, Galectin-3 is more specific in differentiating PTC from benign and non-neoplastic lesions [40]. Both markers, along with negative CD56 and TPO, are also effective in differentiating the classic variant of PTC, which shows strong and diffuse positivity, from the follicular variant PTC (FVPTC), which has more variable expression [40]. Loss of thyroid TPO and CD56 expression is characteristic of PTC; normal thyroid tissue and benign lesions are typically positive for TPO and CD56 [41,42]. Thus, these markers are valuable negative markers in malignant cases. The loss of anti-TPO IHC expression is a moderately sensitive and highly specific marker for identifying tumors with PTC-like nuclear features [41].

Another two diagnostic markers for both PTC and FTC are HBME-1 and trophoblast cell surface antigen-2 (Trop-2), which have recently emerged as a powerful combination for differentiating between malignant and benign lesions [43,44]. HBME-1 is a glycoprotein that binds monoclonal antibodies and is used in diagnosing thyroid and mesothelial malignancies [42,43]. Trop-2 is also another glycoprotein and is expressed in some epithelial cancers, and can also be a target for specific therapy [43,44].

HBME-1 is a highly specific marker for malignancy, with studies showing up to 98% specificity [43]. It is positive in the vast majority of PTC and FTC but is rarely expressed in benign lesions like FA and multinodular goiter [43]. Trop-2 is a highly sensitive marker, with a strong membranous staining pattern being characteristic of PTC and FTC. However, its specificity is lower than HBME-1, as it can be expressed in some benign lesions, particularly oncocytic adenomas [43]. Due to their complementary strengths, a panel combining HBME-1 and Trop-2 can achieve a sensitivity and specificity of up to 98% for malignancy [44].

In addition to the diagnostic markers discussed above, there are prognostic and proliferative markers that can be assessed by IHC as well. These markers help predict the clinical course of the disease, including risk of recurrence and mortality. Of prime importance is E-cadherin, a critical cell adhesion molecule, where the loss of its expression is a significant indicator of aggressive tumor behavior [45]. A meta-analysis confirmed that negative E-cadherin expression is associated with an increased susceptibility to thyroid cancer and correlates strongly with lymph node metastasis, poor differentiation, and advanced TNM stage [45]. Furthermore, studies have shown that lower E-cadherin staining intensity is significantly and inversely correlated with the presence of metastases, making it a powerful prognostic factor [46]. On the contrary, Ki-67, a well-established proliferation marker, has yielded conflicting results [45,46]. Many studies report a very low Ki-67 labeling index across both benign and malignant thyroid lesions, suggesting its limited prognostic value considering that PTC is often a low-proliferation tumor [46]. Other studies on prognosis have identified a high Ki-67 index (>11%) as a strong predictor of cancer-related mortality, suggesting its potential value in identifying the small subset of patients with poor prognosis [47].

Other prognostic IHC markers include *p53*, a well-known tumor suppressor gene surrogate marker, where its expression by IHC has been linked to a higher risk of recurrence [48]. However, its significance as an independent predictor is debated [49].

Recently, metallothionein (MT) and Minichromosome Maintenance Proteins-2 (MCM2) have shown value in distinguishing benign from malignant tumors, but do not appear to correlate with tumor aggressiveness [46]. These markers are potentially promising, and their practical usefulness remains to be seen.

The need for specific and sensitive markers for PTC continues despite the fact that the diagnosis of PTC is usually easy and straightforward. However, some cases pose true diagnostic challenges; in particular, the FVPTC (Figure 3).

The recent improvements in the IHC methodology provide a quick and cost-effective evaluation of some molecular and genetic surrogate markers with an acceptable accuracy, most importantly for *BRAF* mutations, but also for *RAS* and *TERT* mutation analysis [50,51,52,53].

*BRAF* mutations are most frequently seen in PTC, particularly *BRAF*:p.V600E, which is commonly diagnosed by IHC [50,51,52,53,54,55]. It is generally accepted that the presence of the *BRAF*:p.V600E mutation indicates a worse outcome even in microcarcinomas, as it is associated with extra-thyroidal extension, more prominent cytological nuclear features, and greater recurrence risk [54]. For instance, a study on a Mexican mestizo population with PTC revealed that *BRAF:*p.V600E, denoted as *BRAF*:c.1799T>A, may affect diagnosis and prognosis [56]. However, such claims were challenged by Kure et al., whose results pointed to an age-dependent pattern in expression of *BRAF*:p.V600E in PTC, and it is the increasing age rather than the mutation itself that is related to the poor outcome [55].

*TERT* mutations, on the other hand, seem to be more well-recognized by experts as an indicator of poor prognosis with little to no objection to such claims [57,58].

Certain PTC histological variants, like the diffuse sclerosing and the cribriform morular variants, have been detected by IHC. IHC was able to detect the anaplastic lymphoma kinase (ALK) rearrangements in the diffuse sclerosing subtype and nuclear translocation due to activated Wnt signaling in the cribriform morular subtype. This is of clinical significance, especially for the cribriform variant, as it can be associated with mutations of adenomatous polyposis coli (*APC*) of the familial adenomatous polyposis (FAP) syndrome [36].

One of the major strains of molecular and genetic analysis in thyroid disorders is the expensive nature of these tests and their occasional lack of specificity. Therefore, we strongly believe that the exploration of more cost-effective IHC markers for additional surrogate proteins of these abnormalities will continue. The acceptable accuracy of *BRAF*:p.V600E expression by modern IHC techniques was very encouraging for researchers in this regard. The most relevant IHC markers of these are shown in Table 4.

### 4.2. IHC Markers in Follicular Thyroid Carcinoma

The diagnosis of FTC is a clinical challenge since it cannot be differentiated from its benign counterpart, FA, by FNA alone, since it requires the histopathologic identification of capsular and/or vascular invasion for a definitive diagnosis (Figure 1C,D) [34]. The utilization of IHC can help distinguish between FTC and FA [34].

While no single marker is perfect in differentiating FTC from its benign counterpart, a panel of IHC markers can significantly increase diagnostic accuracy. Similar to their role in discriminating PTC from benign lesions, HBME-1, TROP-2, and TPO have been helpful in differentiating benign from malignant follicular-derived thyroid lesions [41,42,43,44]. A recent IHC marker, family with sequence similarity 172 member A protein (FAM172A), is highly expressed in FTC tissues and cell lines and has been identified as a novel tumor promoter [59]. Functional studies show that FAM172A enhances cell proliferation, migration, and invasion through the Erk1/2 and C-Jun N-terminal kinase (JNK) subfamilies of the mitogen-activated protein kinase (MAPK) signaling pathway [59]. It has high sensitivity (92%) and specificity (80%) in excised tissues, and shows similar utility in FNA samples. Therefore, IHC expression of FAM172A stands out as a promising diagnostic tool [59]. In addition, this tumor promoter protein expression was found to be a potential therapeutic target for FTC [59].

### 4.3. IHC in Oncocytic Carcinoma

A quite distinctive feature of oncocytes is the abundance of mitochondrial organelles in the cytoplasm, which gives its distinctive eosinophilic granular appearance on routine Hematoxylin and Eosin stains [34]. Therefore, the utility of the human mitochondrial antibody (HMA) reveals the presence of oncocytic cells, regardless of their malignant nature. This is helpful in diagnosing follicular-patterned neoplasms since oncocytic cells may sometimes be misdiagnosed as the oncocytic variant of PTC, in which HMA will be negative. This is useful in differentiating an oncocytic neoplasm from the oncocytic variant of PTC [34].

Oncocytic tumors demonstrate a different pattern of Tg staining as opposed to PTC and FTC; perinuclear dense granular deposits are what distinguish them from the other follicular-derived neoplasms [36]. The same malignant-discriminating biomarkers useful in diagnosing other DTC subtypes are also identified in OTC, namely Galectin-3, HBME-1, and CK19 [34]. However, these markers were found to be unreliable to differentiate between oncocytic adenoma versus carcinoma [34]. To distinguish between OTC and oncocytic adenoma, a study by Lori et al. suggested utilizing the proliferation marker Ki-67 and the cell cycle marker cyclin D1 by IHC [60]. Moreover, the Close homolog of L1 (CHL1) is a new emerging biomarker introduced by Li et al., demonstrating promising results in distinguishing OTC from its benign counterpart [61].

## 5. Molecular and Genetic Biomarkers of Differentiated Thyroid Carcinoma

Molecular assays are essentially used for indeterminate thyroid nodules (Bethesda III/IV) and advanced or recurrent cases of thyroid cancer to refine risk and guide management [62,63]. In the United States, there are three leading genetic assays for thyroid cancers, namely Afirma Genomic Sequencing Classifier (GSC), ThyroSeq v3, and ThyGeNEXT/ThyraMIR (MPTX) [64,65]. All use FNA specimens but differ in molecular targets and testing methodology [65]. Afirma GSC is an mRNA expression–based test that determines whether a thyroid nodule is “negative” or “suspicious”. ThyroSeq v3 relies on dual DNA- and RNA-based next-generation sequencing (NGS) panels, screening for over 100 thyroid-specific mutations and gene fusions. Furthermore, MPTX not only screens for dual DNA and RNA targets, but also microRNA expression [66]. All these platforms are particularly effective as “rule-out” rather than “rule-in” tools [64].

In advanced and recurrent cases of thyroid cancer, there is advocacy to utilize comprehensive multi-platform DNA and RNA NGS on tumor tissue [62,65,67]. If this is not feasible, three-tiered molecular testing can be carried out. First, perform molecular testing for common driver genes, especially *BRAF*, preferably by direct sequencing. Second, for patients with negative results, gene fusions can be explored by using RNA sequencing [67]. Then, as with reflex testing, if no driver alteration is detected, comprehensive DNA/RNA sequencing is recommended [67]. If sequencing is not available, other molecular techniques can be used to identify key genetic alterations [68]. For instance, the polymerase chain reaction (PCR) can be used to detect targeted DNA variants. For identifying gene fusions, methods like reverse transcription PCR (RT-PCR) and fluorescence in situ hybridization (FISH) are commonly used [68].

**Figure 4:**
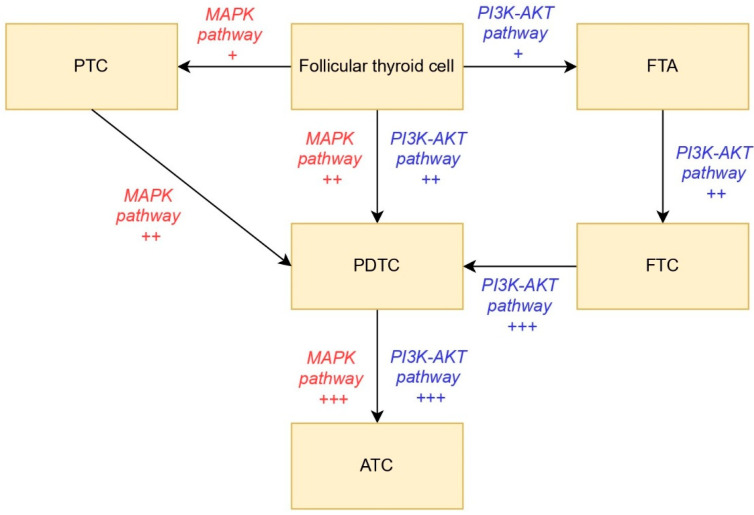
Schematic representation of thyroid tumor progression from follicular thyroid cells to adenoma, PTC, FTC, PDTC, and ATC. The diagram highlights the relative activation of the *MAPK* (red) and *PI3K*–*AKT* (blue) pathways at each stage. “+” symbols indicate increasing pathway activity. Adapted conceptually from prior models with original formatting [69,70]. Reproduced with permission of Springer Nature from [69]; permission conveyed through Copyright Clearance Center, Inc. Abbreviations: PTC, papillary thyroid carcinoma; FTC, follicular thyroid carcinoma; FTA, follicular thyroid adenoma; PDTC, poorly differentiated thyroid carcinoma; ATC, anaplastic thyroid carcinoma; *MAPK*, mitogen-activated protein kinase; *PI3K*–*AKT*, phosphatidylinositol 3-kinase–AKT signaling pathway.

**Figure 5:**
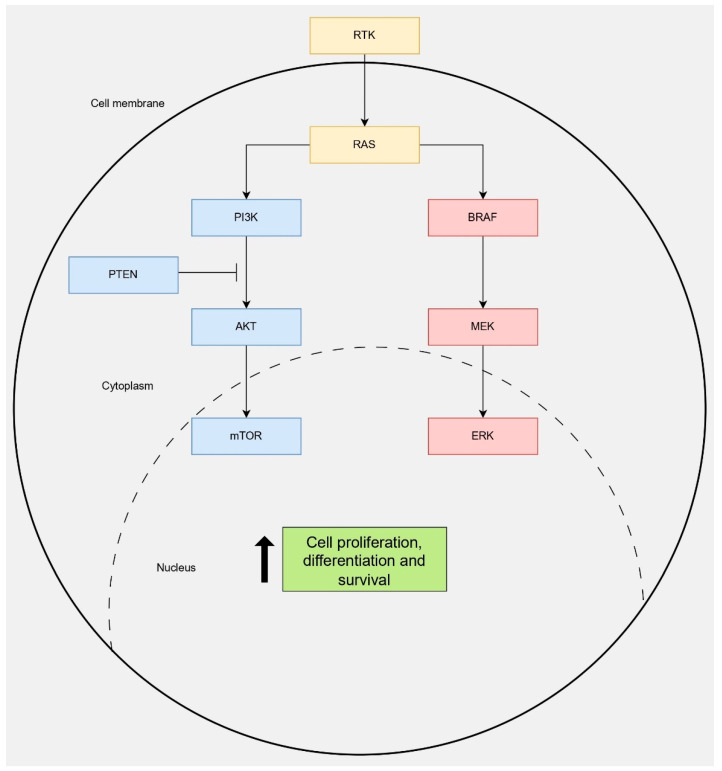
Overview of *MAPK* and *PI3K*/*AKT* signaling pathways in thyroid tumorigenesis. Mutations in genes such as *BRAF*, *RAS*, *PIK3CA*, and *PTEN* dysregulate these cascades, promoting cell proliferation, differentiation, and survival. The dotted line represents schematic nuclear boundary, drawn to indicate that the signaling pathways activated in the cytoplasm ultimately exert their effect within the nucleus. Abbreviations: RTK, receptor tyrosine kinase; RAS, rat sarcoma viral oncogene homolog; PI3K, phosphoinositide 3-kinase; AKT, protein kinase B; PTEN, phosphatase and tensin homolog; mTOR, mechanistic target of rapamycin; BRAF, B-Raf proto-oncogene; MEK, mitogen-activated protein kinase; ERK, extracellular signal-regulated kinase.

The genetic landscape of thyroid tumors is highly diverse. Nevertheless, the *MAPK* and phosphoinositide 3-kinase/protein kinase B (*PI3K*/*AKT*) signaling pathways are commonly implicated in thyroid tumorigenesis as demonstrated in Figure 4 and Figure 5 [69,70]. Additional molecular markers relevant to each subtype of the DTC are highlighted in the following subsections.

### 5.1. Molecular and Genetic Markers of PTC

In addition to their diagnostic value, which was alluded to in the aforementioned IHC section, currently, well-established evidence documenting the prognostic impact of molecular markers across subtypes of thyroid cancer remains limited. A meta-analysis by Vuong et al. published in 2017 attempted to assess the prognostic significance of selected molecular markers on the mortality and recurrence rates in patients with PTC [71].

Interestingly, two promoter mutations, hg19:chr5:1,295,250G>A (aka C250T or *TERT*:c.-146C>T) and hg19:chr5:1,295,228G>A (aka C228T or *TERT*:c.-124C>T) in the *TERT*, occur in 10% of cases with PTC [72]. Moreover, *TERT* promoter mutations have been found in several other thyroid cancer subtypes, such as FTC, poorly differentiated thyroid carcinoma (PDTC), and ATC [73]. These mutations are more prevalent in aggressive subtypes such as PDTC and ATC, accounting for frequencies of about 20–50% and 30–70% in these thyroid lesions, respectively [73,74].

These promoter mutations were found to be significantly associated with unfavorable outcomes in patients with PTC [71]. Among patients with *TERT* promoter mutations, the risk of PTC-specific mortality was 7.64 times higher, and the risk of recurrence was 2.98 times higher compared to those without the mutation [71]. Consequently, *TERT* promoter mutations proved to be a reliable and independent molecular marker to predict the prognosis in patients with PTC harboring these mutations [71,75]. Of note, *TERT* promoter mutations are believed to be less frequent and subclonal in PTC compared with other subtypes of thyroid cancer [76]. Although this limits the integration of *TERT* promoter mutations into PTC risk stratification strategies, they remain highly informative in selecting high-risk cases.

Beyond the promoter mutations in *TERT*, the *BRAF*:p.V600E is the most frequent mutation in the carcinogenesis of PTC (Figure 4 and Figure 5) [77,78]. This marker is considered highly specific for thyroid cancer, albeit poorly sensitive [64]. *BRAF*:p.V600E occurs in around 43–88% of PTC cases [78]. The presence of *BRAF*:p.V600E is considered a highly diagnostic marker for classical PTC, and even more powerful for the tall cell variant of PTC [79,80]. In this context, a recent study, published in 2025, by Brunfield and colleagues demonstrated a significant association between *BRAF*:p.V600E and the phenotypic subtype of PTC [81]. Specifically, all the screened cases showing tall cell variants of PTC harbored *BRAF*:p.V600E compared to 88% and 38% of cases displaying the classical PTC and extensive follicular growth of PTC, respectively [81]. 

Interestingly, indolent thyroid neoplasms like FAs and encapsulated follicular-patterned neoplasms typically lack the *BRAF*:p.V600E molecular alteration and show *RAS*-like molecular profile [80]. The progression to infiltrative and aggressive forms of PTC is marked by the shift from a *RAS*-like to a *BRAF*-like signature, usually by the acquisition of the *BRAF*:p.V600E mutation [80].

The prognostic impact of *BRAF* mutations has been extensively studied, and the results are controversial [82]. For instance, a meta-analysis by Li C. et al., published in 2013, pooled 32 studies including 6372 patients with PTC, and found that *BRAF*-mutated PTC was significantly associated with larger tumor size, lymph node metastasis, extrathyroidal extension, advanced tumor stage, multifocality, tall cell variant histology of PTC, classic variant of PTC, and absence of a capsule [83]. Despite the potential prognostic implications of *BRAF*:p.V600E proposed by such meta-analyses, the nature of the screened studies was retrospective in nature with potential selection bias. Therefore, caution should be advised when seeking medical decisions regarding *BRAF*:p.V600E-related PTC [83].

More recent evidence has questioned the *BRAF*:p.V600E prognostic value. In 2017, a meta-analysis by Vuong et al., investigated the prognostic impact of *BRAF*:p.V600E mutations based on 35 studies, containing 17,732 patients with PTC [71]. They found out that patients with PTC carrying *BRAF*:p.V600E exhibited a significantly higher risk of disease recurrence compared to those without the mutation (63%). Nevertheless, subgroup analysis revealed that the prognostic value of *BRAF*:p.V600E was exclusive to short- and medium-term follow-up and diminished with longer-term outcomes in PTC. Furthermore, the presence of the *BRAF*:p.V600E mutation did not significantly impact mortality rates in patients with PTC [71].

Another recent meta-analysis by Wei et al., published in 2022, associated the *BRAF*:p.V600E mutations in PTC with more aggressive tumor features [84]. These features encompassed increased multifocality, vascular invasion, extrathyroidal extension, and advanced TNM stage (III–IV). Although this study reinforced the association between *BRAF*:p.V600E and certain aggressive tumor features, it did not directly assess the recurrence or mortality outcomes in PTC cases. Consequently, although *BRAF*:p.V600E may indicate a higher-risk tumor profile, its value as a long-term prognostic marker currently remains uncertain [84]. Given this uncertainty, prospective future studies specifically addressing this will be needed.

More recently, in 2025, a study by Brumfield et al. attempted to elucidate the impact of harboring *BRAF*:p.V600E in around 300 patients with PTC [81]. Intriguingly, they showcased that *BRAF*:p.V600E alone does not predict recurrence in PTC lacking high-risk histological features, limiting its value as an independent risk stratification marker [81].

On another note, the co-existing mutations in the PI3K/AKT/mTOR pathway alongside *BRAF*:p.V600E in patients with PTC demonstrated a significantly higher PTC-specific mortality rate (Figure 4 and Figure 5) [85].

The co-occurrence of *TERT* promoter mutations with *BRAF*:p.V600E has been shown to aggravate the trajectory of the disease [71,86,87]. Vuong et al. demonstrated that PTC harboring *BRAF*:p.V600E and *TERT* mutations had significantly worse mortality compared to those with a sole *BRAF* mutation [71]. Notably, the outcomes were similar between *TERT*-only and dual-mutant PTCs, which consisted of *TERT* and *BRAF*:p.V600E. This further reinforces *TERT* as an independent and standalone prognostic marker of PTC, regardless of the *BRAF* status [71].

Several multi-kinase inhibitors (MKIs) such as Lenvatinib and Sorafenib are considered the standard first-line therapies for radioactive iodine (RAI)-refractory DTC [88,89]. However, MKIs lack target specificity, and patients often experience adverse effects arising from their off-target actions. More recently, increasing attention has been directed towards biomarker-driven therapy for DTC [88]. In particular, *BRAF*:p.V600E has been investigated as a therapeutic target in patients with RAI-refractory DTC harboring this mutation. Dabrafenib, a selective *BRAF* inhibitor, has been evaluated in this context. Clinical studies of dabrafenib, either as monotherapy or in combination with the MEK inhibitor (Trametinib), have reported objective response rates of approximately 30–35% in patients with *BRAF*:p.V600E–mutated, RAI-refractory DTC [88,90]. Notably, the combination of dabrafenib and trametinib was not found to be superior to dabrafenib monotherapy in this setting [88,90].

Another extensively studied prognostic marker is *RAS*. Mutations in *RAS* are estimated to occur in 24.36% of typical PTC [91]. Higher prevalence of *RAS* mutations was recorded in FVPTC (55.2%) and encapsulated FVPTC (47.92%) [91].

The prognostic implications of *RAS* mutations in PTC have been debated. A study by Hara et al. in 1994 noted that the occurrence of *NRAS* mutations in PTC was a significant and independent predictor of mortality and recurrence [92]. Nonetheless, these were recently debated by Vuong et al., due to the limited number of studies documenting the mortality and recurrence rates of *RAS* mutations in PTC cases [71]. Previous observations of PTC’s aggressive behavior might have been influenced by other coexisting mutations, specifically in the *TERT* promoter area. Consequently, for *RAS* mutations to have a prognostic impact, they must be considered alongside additional molecular markers [93]. Further studies are needed to establish the prognostic impact of sole *RAS* mutations on PTC [71,92].

Despite their rarity, oncogenic chromosomal rearrangements have been described in thyroid cancer, especially in pediatrics [94]. *RET*/*PTC* chromosomal rearrangements were first discovered in PTC and are strongly attributed to radiation exposure, especially in young patients [72,74]. Estimated to occur in 10–30% of cases, these clonal *RET*/*PTC* rearrangements exhibit a notable specificity to radiation-induced PTC [72,93]. In contrast, low-level or non-clonal *RET*/*PTC* rearrangements have been detected in more benign thyroid conditions, like Hashimoto thyroiditis and thyroid hyperplasia [74]. There are around 20 different *RET*/*PTC* alterations, depending on the genomic region fused to the *RET* gene [93]. *RET*/*PTC1* and *RET*/*PTC3*, corresponding to *RET-CCDC6* and *RET*-*NCOA4*, respectively, account for 90% of all *RET* rearrangements [72]. According to the meta-analysis by Voung H. et al., the prognostic value of *RET*/*PTC* rearrangements was inconclusive, necessitating further investigations [71]. *RET* fusions can be used as therapeutic targets in advanced, metastatic, or RAI-refractory DTC [89]. Selective *RET* inhibitors such as Selpercatinib and Pralsetinib are approved therapeutic options in such patients [88,89].

Neurotrophic tyrosine receptor kinase (*NTRK*) rearrangements include fusions of the *NTRK1* and *NTRK3* genes with various partner genes, resulting in constitutive activation of TRK signaling pathways [95]. The most common fusions in PTC are *ETV6*–*NTRK3* and *TPM3*–*NTRK1* [74]. Despite being rare in adults, they occur more frequently in pediatrics and radiation-induced PTC [95]. *ETV6*–*NTRK3* has been commonly linked to radiation-induced PTC and conventional PTC with follicular growth, and FVPTC [74]. The prognostic impact of *NTRK* alterations currently remains unclear [95]. Some findings indicated that *NTRK3*-rearranged PTCs, compared to *NTRK1*-rearranged PTCs, exhibit follicular-like growth patterns, follow a less aggressive clinical course, and are associated with longer progression-free survival [94]. Noteworthy, *NTRK* fusions have actionable value, with Larotrectinib and Entrectinib being recommended in the treatment of solid tumors with this fusion [88,89].

*ALK* rearrangements occur through the fusion of *ALK* with another gene partner [96]. *STRN* and *EML4* have been predominantly described as *ALK*-fusion partners in PTC [96]. *STRN*-*ALK* has been estimated to account for 0–7% of each pediatric- and adult-onset PTC [97,98]. Some evidence presented this fusion with an advanced stage of PTC [98]. However, until now, no prognostic effect for this rearrangement has been observed in cases with PTC [94,98].

Rearrangements involving the *THADA* gene, particularly the *THADA-IGF2BP3* fusion, have been described in FA, non-invasive follicular thyroid neoplasm with papillary-like nuclear features (NIFTP), and PTC [99,100,101]. Of the PTCs, both the FVPTC and classic variant of PTC have been shown to harbor the *THADA-IGF2BP3* fusion. Both of which frequently exhibited encapsulation [99,100]. These fusions are identified in around 2% of indeterminate thyroid nodules (Bethesda III–IV) with more than 80% risk of malignancy [99,100]. Importantly, *THADA*-*IGF2BP3*-positive tumors exhibit indolent biological behavior without the occurrence of lymph node metastasis or extrathyroidal extension [99,100]. Patients with thyroid tumors harboring *THADA-IGF2BP3* fusion showed no disease progression or recurrence on short- to mid-term follow-up [99,100]. Prognostically, the *THADA-IGF2BP3* fusion is considered a potential marker of low-risk cancer potential.

Certain immune and epigenetic markers have emerged with potential significance in PTC. The overexpression of programmed death-ligand 1 (*PD-L1*) in cases with PTC correlated with a higher recurrence risk than those with *PD-L1*-negative tumors [102]. Likewise, several non-coding RNAs have been shown to have plausible prognostic value in PTC. A systematic review identified the high expression of *miR-221*, *miR-222*, and *miR-146b* as strong predictors of PTC recurrence [103].

### 5.2. Molecular and Genetic Markers of FTC

Mutations in the *RAS* genes and *PAX8-PPARγ* rearrangements are characteristic molecular alterations in FTC (Figure 4 and Figure 5) [104]. Therefore, these *RAS* mutations can be utilized as diagnostic markers for FTC [82]. There are three *RAS* isoforms, namely *HRAS*, *NRAS*, and *KRAS,* that define the follicular-patterned thyroid neoplasms, which include FTC, encapsulated FVPTC, and FA [105]. The *RAS* mutations are common in FTC and FA, accounting for 30–50% and 20–40% of these lesions, respectively [74,106]. Among these, the frequently mutated gene in follicular-patterned thyroid lesions is *NRAS*, particularly mutated at codon 61, followed by *HRAS*. However, the presence of *RAS* mutations alone in a thyroid lesion is not detrimental to its malignancy [104]. Rather, thyroid lesions with *RAS* mutations can exhibit a significant tendency to develop follicular-patterned carcinoma [105].

The prognostic effect of *RAS* mutations in FTC has been explored in a meta-analysis by Goh et al. in 2022 [107]. They found out that the presence of *RAS* mutations was significantly associated with distant metastases in FTC, with a relative risk of 2.07 [107]. No significant associations were found between *RAS* alterations and FTC-specific mortality, lymph node metastases, and tumor size [107].

*PAX8-PPARγ* rearrangements emerge due to the fusion of the *PAX8* gene to the *PPARγ* gene due to a t(2;3)(q13;p25) translocation [108]. This rearrangement has been described in 30–60% and around 38% cases of FTC and FVPTC, respectively [108]. To a lesser extent, *PAX8-PPARγ* translocations have been described in OTC (5%), FA (2–10%), and PTC (up to 1%) [109]. Some evidence suggested the propensity of *PAX8*/*PPARγ*-positive FTCs to angioinvasion as an indirect method for assessing tumor aggressiveness [104]. Nevertheless, the current data are limited to the use of *PAX8*/*PPARγ* as a prognostic factor for PTC [104].

The *PI3K*/*AKT* pathway is another key culprit in the development of thyroid cancers (Figure 4 and Figure 5) [110]. Triggered by activating mutations in *PIK3CA* and *AKT1,* as well as inactivation of *PTEN*, several thyroid tumors can emerge, including FA, FTC, PDTC, and ATC [110] (Figure 4 and Figure 5). The *PI3K*/*AKT* pathway serves as a key driver of invasion, metastasis, and disease progression in FTC [69]. Genetic alterations in this pathway are associated with poor prognosis, especially in metastatic FTC, and may predict dependence on *PI3K*/*AKT* signaling for tumor maintenance [69].

The inactivation of *PTEN,* normally serving as a negative regulator of the *PI3K*/*AKT* pathway (Figure 5), promotes thyrocyte proliferation and survival, as commonly seen in Cowden syndrome [111]. Both somatic and germline mutations in *PTEN* contribute to the development of thyroid tumors [110,112]. Somatic mutations in *PTEN* have been implicated in FA, PTC, and ATC [95]. The germline mutations in *PTEN* predispose patients with Cowden syndrome to FTC development [110,113].

### 5.3. Molecular and Genetic Markers of OTC

Despite sharing the same follicular thyroid cell origin, OTC exhibits a unique genetic profile compared to the other subtypes of DTC [114]. This distinctive mutation signature includes alterations in both nuclear and mitochondrial DNA, as well as global genomic chromosomal loss of heterozygosity (gLOH) [115].

Unlike the other subtypes of DTC, *RET*/*PTC*, *PAX8*/*PPARγ* rearrangements, *RAS*, and *BRAF* mutations are infrequently detected in OTC [116]. On the other hand, *TP53* has been raised as a potential diagnostic marker for oncocytic tumors [17]. This is because mutations in *TP53* were more common in OTC than in other thyroid cancers, accounting for 42% of OTC cases [17]. Furthermore, *TERT* promoter mutations have been linked to greater invasion potential, documented in 32% of highly invasive OTC compared to 5% of minimally invasive OTC [17].

As for the mutations in the mitochondrial genome, they are considered a hallmark of oncocytic tumors [17,117,118]. Oncocytic thyroid neoplasms, whether adenoma or carcinoma, harbor a “common deletion” of 4977 bp in a homoplasmic configuration, both of which acquire the “common deletion” at great frequency, with OTC showing the highest burden [17]. Additionally, mutations in mitochondrial complex I of the electron transport chain subunit have been identified in 26% of oncocytic thyroid neoplasms, significantly exceeding other non-oncocytic tumors [118]. These findings underscore the association of oncocytic thyroid neoplasms with mitochondrial abnormalities. Therefore, the mitochondrial abnormalities can support oncocytic features of thyroid tumors but are not solely diagnostic of malignancy since benign adenomas share these features [17].

In addition to mitochondrial alterations, OTC is characterized by global chromosomal aneuploidy and gLOH [115]. Several studies have shown a widespread near-haploid status in OTC, reflecting multiple losses in various chromosomes, with the selective retention or duplication of others. This feature is characteristic of oncocytic neoplasms and is not found in other subtypes of DTC [118]. To date, approximately ten chromosomes have been frequently lost in OTC, specifically chromosomes 1–4, 6, 8–9, 11, 14–15, and 21–22 [118]. Another four chromosomes, 5, 7, 12, and 20, have consistently either been retained or amplified [17,117,118].

Notably, selected gains in chromosomes 12, 19, and 20 and loss of chromosome 22 have been linked to high recurrence frequency and increased mortality, respectively [74]. Cases of OTC exhibiting diploidy and limited chromosomal aberrations had minimal invasion and more favorable outcomes [115]. In contrast, excessive chromosomal losses and widespread gLOH in OTC have been described in aggressive cases with worse outcomes [115]. Table 5 gives a summary of these markers in DTC.

### 5.4. Molecular Imaging Biomarkers in DTC

In addition to the tumor specimens-based molecular biomarkers of DTC, imaging biomarkers are frequently used in the diagnosis, staging/restaging, and prognosis of all subtypes of DTC [63,119,120]. Among these biomarkers are the RAI isotopes I-131, I-123, and I-124, which provide important diagnostic and prognostic information. The imaging modalities used in the case of I-131 and I-123 include the conventional gamma camera or a hybrid system combining a gamma camera with CT (SPECT/CT), while, in the case of I-124, a positron emission tomography (PET) scanner combined with CT (PET/CT) or MRI (PET/MRI) is used [120].

Since most DTCs are at least initially iodine-avid owing to their expression of the sodium iodide symporter (NIS), RAI enables the detection of disease sites throughout the body that can be treated by surgery, therapeutic doses of I-131, or external beam radiation. RAI concentration at disease sites generally portends a good prognosis, both because these sites are amenable to I-131 therapy and because the NIS expression is indicative of tumor cell differentiation [63,119,120]. In contrast, loss of NIS expression/RAI uptake indicates tumor cell dedifferentiation and, hence, worse prognosis [63,119,120].

Fluorodeoxyglucose (FDG) is another imaging biomarker that has been shown to be useful in the detection of non-iodine-avid persistent or recurrent disease and for prognostication with imaging performed using PET/CT or PET/MRI scanners. Most iodine-avid tumor sites show no or low FDG uptake, and this generally indicates a good prognosis [119,120]. However, with cell dedifferentiation, a so-called “flip-flop” occurs where lesions become non-iodine-avid but FDG-avid [121]. This FDG avidity enables the detection of occult disease based on elevated Tg in the face of negative whole body RAI scans with reported sensitivity and specificity as high as ~93% and 84%, respectively, based on a meta-analysis of six studies including 165 patients who underwent FDG-PET/CT [122]. Furthermore, studies have shown that patients with a negative whole body iodine scan and elevated Tg, who are FDG-positive, have a worse prognosis in terms of overall, progression-free, or disease-free survival compared with those who are FDG-negative [120,123]. For example, Vural et al. in a study of 105 patients with a negative I-131 whole body scan and elevated Tg followed up for a mean duration of 3.8 years after FDG-PET showed that only 10/30 patients (33%) with FDG-negative scans progressed or had unsuppressible Tg (>1 ng/mL) under TSH suppression therapy with no incident of disease-specific death [123]. In contrast, 44/75 patients (~59%) with FDG-positive scans were alive with disease, with an additional 6 patients (8%) who died of disease-specific conditions [123].

Additional imaging biomarkers of DTC include 18F-tetrafluoroborate, an anion analog of iodine and a PET tracer for imaging the human NIS [124], as well as Gallium-68 prostate-specific membrane antigen (Gallium-68-PSMA). While PSMA is mainly expressed in prostate carcinoma, it is also expressed in the neovasculature of several tumors, including dedifferentiated thyroid carcinoma [125]. More detailed discussion of the utility, advantages, and limitations of these biomarkers is beyond the scope of this review.

## 6. Limitations of This Narrative Review

The review presented herein is a narrative rather than a systematic review or meta-analysis. A narrative review is susceptible to bias because it does not follow a rigorous, predefined protocol like systematic reviews, potentially resulting in reduced transparency and greater bias. Moreover, the decision to include or exclude studies in a narrative review is sometimes subjective, which can lead to skewed conclusions. The potential inclusion of low-quality studies in narrative reviews can also contribute to inaccurate conclusions. While low-quality studies may also be included in systematic reviews, their inclusion is clearly acknowledged so that the reader can consider their impact on the overall review findings and understand the limitations of the review. Since our review is a narrative one, a systematic assessment of the quality of the evidence or papers was not required and hence, not performed.

## 7. Conclusions and Future Directions

DTC is very common, and the incidence is on the rise worldwide. The burden of this cancer is considerable and consequently consumes a sizable fraction of total health expenditure. Although the majority of these carcinomas are PTCs and accordingly have an excellent prognosis, many patients with thyroid nodules undergo unnecessary surgery, which inflicts unnecessary risk on patients and adds cost to healthcare systems. Thyroid nodules evaluated by FNA, with its recent advancement in technique and standardized reporting system, provided significant improvements in triaging guidelines. Many of the clinical and histopathological parameters, which impact prognosis, are well-known and extensively studied. A few exceptions of these are still subject to debate, and future large-scale studies are required. Thus far, evaluation of capsular and lymphovascular invasion, the hallmark of carcinoma in follicular-patterned thyroid lesions, is impossible on FNA cytology. Therefore, the search for better tools to help navigate this dilemma continues. Hopefully, future prospective analyses will be fruitful. The thyroid IHC landscape is evolving rapidly, with more accurate diagnostic and prognostic markers on the horizon. Thyroid organ-specific markers, such as Tg, PAX8, and TTF-1, are currently in routine use in the majority of laboratories that perform IHC. More markers are expected to be discovered that may be helpful. Improvements in the IHC detection of proteins as surrogate markers for molecular and genetic alterations are expected to be revolutionary. Future validation studies on these new proteins may refine the sensitivity and specificity of these IHC markers as a cost-effective approach. The molecular and genetic alterations in DTC, such as *BRAF:*p.V600E, *RAS* mutations, *TERT* promoter mutations, and *RET*/*PTC* rearrangements, provide valuable diagnostic and prognostic insights into these carcinomas. Detection of these alterations is currently available for clinical as well as research use. There are multiple detection techniques, each with its own pros and cons; however, NGS is becoming the frontrunner due to its robust qualities. Innovations in NGS and other laboratory schemes have helped polish risk stratification, facilitating precision medical approaches. It is anticipated that future studies will focus on validating emerging biomarkers in DTC with emphasis on the improvement of the diagnostic and prognostic usefulness of these assays. Improving patients’ outcomes will be the crucial determinant.

## Figures and Tables

**Table 1:** Various clinical parameters determining prognosis in DTC.

Clinical Parameter	Prognostic Significance
Advanced age at diagnosis	Worse prognosis [1,14]
Hashimoto’s thyroiditis	Higher risk of developing thyroid carcinomas [3,12]
Graves’ disease	Higher risk of developing thyroid carcinomas [12,13]
Multinodular goiter	Higher risk of developing thyroid carcinomas [12,13]
Obesity	Higher risk of developing thyroid carcinomas [1,15]
Family history of thyroid cancer	Higher risk of developing thyroid carcinomas [1,15]
Distant and lymph node metastasis at diagnosis	Worse prognosis [14,15,18,19,20]
History of radiation	Uncertain behavior [14,15,16,17,18]

**Table 2:** This table summarizes the most important histopathological parameters in PTC and their prognostic significance.

Histopathological Parameter	Prognostic Significance
Tall cell, hobnail, columnar, diffuse sclerosing, solid, trabecular, and insular variants	Worse prognosis, although one 2022 report showed that the solid/trabecular/insular growth does not have prognostic significance by itself [8,24,25,26]
Encapsulated variants	Good prognosis [20,22]
Invasion and extrathyroidal extension	Worse prognosis [20,21]
Central lymph node metastasis (N1a)	No change in clinical prognosis compared to N0 patients [25]
Lateral lymph node metastasis (N1b)	Worse prognosis [25]
Distant metastasis	Worse prognosis [18,19,21,23]
Multifocal disease	Controversial [8,23,24]

**Table 3:** Summary of the most important histological parameters of follicular carcinoma and their prognostic significance.

Histopathological Parameter	Prognostic Significance
Capsular invasion without vascular invasion	Good prognosis [29]
Vascular invasion	Adverse prognosis [30]
Multiple foci of vascular invasion	Adverse prognosis [30]
Lymph node involvement and distant metastasis	Adverse prognosis [5,8,21,31]

**Table 4:** Summary of the most relevant immunohistochemical (IHC) biomarkers used in papillary thyroid cancer (PTC).

IHC Marker	Value	Comment
HBME-1	Diagnostic	Highly specific marker for malignancy with 98% specificity [43]. Very effective when combined with TROP-2 to differentiate malignant from benign lesions, especially in differentiating PTC from nonneoplastic lesions [44]
Tg	Diagnostic	Confirms thyroid follicular cell origin [34,35,36,37,38]
CK19 and Galectin-3	Diagnostic	CK19 is a low molecular weight cytokeratin in epithelial cells. Galectin-3 is a β-galactoside-binding lectin responsible for cell adhesion. Both are used in combination to distinguish PTC from benign lesions [39]
TPO and CD56	Diagnostic	Valuable negative markers for malignancy. Loss of expression of TPO and CD56 is characteristic of PTC, with normal thyroid tissue and benign lesions typically positive for these markers [41,42]
E-cadherin	Prognostic	Proliferative marker, the loss of its expression has been found to be a significant indicator of aggressive tumor behavior, lymph node metastasis, poor differentiation, and advanced TNM stage [45,46]
Ki-67	Prognostic	Proliferative marker with conflicting results, but one study showed that a high Ki-67 index (>11%) is a strong predictor of cancer-related mortality, and therefore, poor prognosis [46,47]
PAX8	Diagnostic	It helps to confirm thyroid origin of cells, but because of low organ specificity, it is recommended to use it in conjunction with other markers [38]
MT and MCM2	Diagnostic	Promising markers differentiating benign from malignant, but no relationship to aggressiveness [46]
BRAF	Prognostic	Some studies indicate that *BRAF*:**p.V600E** mutation is associated with extrathyroidal extension, more prominent cytological nuclear features, greater recurrence risk, and worse outcome [54] (please see the molecular and genetic section for more details)
TERT	Prognostic	An indicator of poor prognosis [57,58] (please see the molecular and genetic section for more details)

Abbreviations: Hector Battifora’s Mesothelioma 1 (HBME-1), Thyroglobulin (Tg), Cytokeratin19 (CK19), metallothionein (MT), and Minichromosome Maintenance 2 (MCM2).

**Table 5:** Summary of molecular markers in differentiated thyroid carcinoma (DTC).

Marker	Subtype(s)	Value	Comment	Citation(s)
*TERT* promoter mutations	PTC	Prognostic	Promoter mutations in *TERT* significantly predict worse outcomes in PTC (≈7.6-fold higher mortality, ≈3-fold recurrence risk). Independent prognostic marker in PTC.	[71,75]
*BRAF*:p.V600E mutation	PTC	Diagnostic and prognostic	Highly specific diagnostic marker for classical PTC. Associated with aggressive tumor features (multifocality, invasion, advanced stage) and higher recurrence in some studies, though its long-term prognostic value is debated.	[79,80,83,84]
*RAS* (*NRAS*/*HRAS*/*KRAS*) mutations	PTC, FTC	Diagnostic and prognostic	Common in follicular-pattern tumors. Diagnostic in FTC (found in ~30–50% of FTC) and prevalent in FVPTC. In the FTC, *RAS* predicts distant metastases. In PTC, prognostic impact is unclear and likely depends on co-mutations.	[71,82,91,107]
*RET*/*PTC* rearrangements	PTC	Diagnostic	*RET*/*PTC* fusions occur in ~10–30% of PTC (especially radiation-induced cases). Highly specific to PTC, but meta-analyses show inconclusive prognostic significance.	[71,72]
*NTRK1*/*3* fusions (e.g., *ETV6*–*NTRK3*, *TPM3*–*NTRK1*)	PTC (pediatric, radiation)	Diagnostic	Rare NTRK gene fusions in PTC, more common in pediatric or radiation-related cases. Prognostic impact is unclear; some data suggest *NTRK3* fusions have follicular-like, less aggressive behavior.	[94,95]
*ALK* gene fusions (*STRN-ALK*, *EML4-ALK*)	PTC	Diagnostic	Uncommon *ALK* fusions in PTC (0–7% of cases). Reportedly seen in advanced-stage tumors, but to date, no clear prognostic effect has been demonstrated.	[94,96,98]
*THADA*–*IGF2BP3* fusion	PTC	Prognostic	Fusion is seen in a minority (~2% of indeterminate nodules) of PTC (often encapsulated FVPTC or classic). Tumors with *THADA*–*IGF2BP3* exhibit indolent behavior (no lymph node metastasis or invasion) and low recurrence, making it a marker of low-risk disease.	[99,100]
*PD-L1* overexpression	PTC	Prognostic	High PD-L1 expression in PTC is linked to higher recurrence risk.	[102]
*miR-221*/*miR-222*/*miR-146b*	PTC	Prognostic	Elevated levels strongly predict PTC recurrence.	[103]
*PAX8*–*PPARγ* rearrangement	FTC	Diagnostic	Common in FTC (~30–60% of cases) (also seen in FVPTC). Serves as a hallmark diagnostic marker of FTC. Some evidence links it to vascular invasion (aggressiveness) in FTC. Limited evidence for prognostic use in PTC.	[104,108]
*PIK3CA* mutation	FTC	Prognostic	Activating PIK3CA mutations stimulate the PI3K/*AKT* pathway. *PI3K*/*AKT* pathway alterations drive FTC invasion and metastasis, indicating poor prognosis in metastatic FTC.	[69,110]
*AKT1* mutation	FTC	Prognostic	AKT1 activating mutations also trigger *PI3K*/*AKT* signaling. Associated with invasive, metastatic behavior in FTC.	[69,110]
*PTEN* loss/mutation	FTC (Cowden), PTC	Diagnostic and prognostic	Loss of *PTEN* (negative regulator of *PI3K*/*AKT*) predisposes to thyroid tumors. Germline *PTEN* mutations (Cowden syndrome) strongly predispose to FTC; somatic *PTEN* alterations occur in thyroid cancers. PTEN loss leads to *PI3K*/*AKT* activation and is linked to aggressive tumor behavior.	[95,110,111,113]
*TP53* mutation	OTC	Diagnostic	*TP53* mutations are much more common in OTC (~42%) than in other thyroid cancers. *TP53* is thus a potential diagnostic marker for OTC.	[17]
*TERT* promoter mutations (OTC)	OTC	Prognostic	*TERT* promoter mutations are more frequent in widely invasive OTC (32%) than minimally invasive ones (5%), correlating with greater invasive potential.	[17]
Mitochondrial DNA alterations (“common deletion”, complex I mutations)	OTC	Diagnostic	Oncocytic tumors characteristically harbor a mitochondrial 4977 bp “common deletion” and frequent complex I gene mutations. These changes underline the oncocytic phenotype (diagnostic of oncocytic neoplasia), though they also occur in benign oncocytic adenomas.	[17,115,118]
Global chromosomal aneuploidy/LOH	OTC	Prognostic	OTC often exhibits near-haploid genomic profiles with widespread chromosomal losses. Extensive loss of chromosomes (e.g., 1–4,6,8–9,11,14–15,21–22) associates with high recurrence/mortality, whereas limited aberrations (diploidy) indicate a more favorable outcome.	[115,117]

Abbreviations: PTC, papillary thyroid carcinoma; FTC, follicular thyroid carcinoma; OTC, oncocytic (Hürthle cell) thyroid carcinoma; FVPTC, follicular variant of papillary thyroid carcinoma; DTC, differentiated thyroid carcinoma; LOH, loss of heterozygosity; mtDNA, mitochondrial DNA; miR, microRNA; PD-L1, programmed death-ligand 1.
